# Treatment of Non-Small-Cell Lung Cancer with Erlotinib following Gefitinib-Induced Hepatotoxicity: Review of 8 Clinical Cases

**DOI:** 10.1155/2012/354657

**Published:** 2012-11-08

**Authors:** Yukihiro Yano, Yoshinobu Namba, Masahide Mori, Yukie Nakazawa, Ayumi Nashi, Shinichi Kagami, Manabu Niinaka, Tsutomu Yoneda, Hiromi Kimura, Toshihiko Yamaguchi, Soichiro Yokota

**Affiliations:** ^1^Department of Thoracic Oncology, National Hospital Organization Toneyama National Hospital, 5-1-1 Toneyama, Toyonaka, Osaka 560-8552, Japan; ^2^Pharmaceutical Department, National Hospital Organization Toneyama National Hospital, Toyonaka, Osaka, Japan

## Abstract

*Objective*. Gefitinib often induces liver damage. A few reports have described that the subsequent administration of erlotinib was associated with less hepatotoxicity, but the safety and efficacy of this treatment are still not fully investigated. Therefore, we evaluated retrospectively the patients with erlotinib following gefitinib-induced hepatotoxicity. *Methods and Patients*. We retrospectively reviewed the medical records between December 2007 and March 2010. The patients were evaluated including the following information: age, gender, histology of lung cancer, performance status, smoking status, epidermal growth factor receptor (EGFR) mutation status, liver metastasis, viral hepatitis, alcoholic liver injury, clinical response, and hepatotoxicity due to EGFR tyrosine kinase inhibitors. *Results*. We identified 8 patients with erlotinib following gefitinib-induced hepatotoxicity. All achieved disease control by gefitinib. Hepatotoxicity was grades 2 and 3 in 3 and 5 patients, respectively. The median duration of treatment with gefitinib was 112.5 days and the median time to gefitinib-induced hepatotoxicity was 51.5 days. The median duration of treatment with erlotinib was 171.5 days. Grade 1 and 2 erlotinib-induced hepatotoxicity was observed in 2 and 1 patient, respectively. *Conclusions*. Erlotinib administration with careful monitoring is thought to be a good alternative strategy for patients who respond well to gefitinib treatment but experience hepatotoxicity.

## 1. Introduction

Lung cancer is the leading cause of cancer death worldwide. For patients with advanced non-small-cell lung cancer (NSCLC), systemic chemotherapy combined with platinum compound and a third-generation agent is considered as standard first-line treatment. On the other hand, gefitinib, one of the epidermal growth factor receptor-tyrosine kinase inhibitors (EGFR-TKIs), is effective, especially in patients with adenocarcinomas who are women, never-smokers and Asian [1].Recently, several studies have shown that NSCLC tumors were highly responsive to the EGFR-TKIs, gefitinib and erlotinib, in patients with somatic mutations of the EGFR gene, such as a point mutation at exon 21 (L858R) or a base pair-deletion at exon 19 (del746_A750) [2–4]. Moreover, an improvement in progression free survival was also seen in NSCLC patients with an EGFR gene mutation who were treated with first-line gefitinib [5, 6]. However, various adverse events, such as skin rash and diarrhea, have been seen following gefitinib treatment and among them, hepatotoxicity is relatively underappreciated. Severely elevated aminotransferase levels occurred in approximately a quarter of gefitinib-treated patients in recent Phase III trials conducted in Japan [5, 6]. In our clinical experience, we have seen hepatotoxicity due to gefitinib, which has resulted in the withdrawal of the treatment. Therefore, it is important that gefitinib-induced hepatotoxicity is managed correctly. The successful management of some cases of gefitinib-induced hepatotoxicity has been reported through the temporary withdrawal of gefitinib or switching the gefitinib administration from daily to every other day [7–9]. However, resumption of gefitinib failed because of worsening hepatotoxicity in two of three cases [7, 8], or an intermittent administration of gefitinib maintained the response for only 8 weeks [9]. Although on the face of it, switching from gefitinib to erlotinib is reasonable because both drugs share a common chemical structure. On the other hand, this very similarity could mean that erlotinib treatment might also cause hepatotoxicity. Nevertheless, a few successful cases have been reported showing that subsequent administration of erlotinib was feasible after gefitinib-induced hepatotoxicity and it was less frequently associated with hepatotoxicity [10–12]. It is desirable for the patients who are maintaining the response to continue the EGFR-TKIs even if drug-induced hepatotoxicity occurs. However, the safety and efficacy of treatment with erlotinib after gefitinib-induced hepatotoxicity are still not fully investigated. Therefore, we evaluated retrospectively all patients who suffered from hepatotoxicity due to gefitinib and subsequently changed their treatment to erlotinib, during an approximately 2-year period at our institution. 

## 2. Patients and Methods

We identified all patients who were treated with gefitinib between December 2007 and March 2010 at Toneyama National Hospital in Osaka, Japan. From these patients, we selected those who discontinued gefitinib treatment and switched to erlotinib because of gefitinib-induced hepatotoxicity. All these patients discontinued their treatment with gefitinib by grade 2 or more of gefitinib-induced hepatotoxicity. The medical records of this subgroup of patients were retrospectively reviewed for the present study as of October 2010, and the following information was retrieved: age, gender, histology of lung cancer, performance status, smoking status, EGFR gene mutation status, liver metastasis, viral hepatitis, alcoholic liver injury, clinical response, and hepatotoxicity due to both gefitinib and erlotinib. We defined hepatotoxicity as the elevation of liver aminotransferase. All toxicities were graded according to the National Cancer Institute Common Terminology Criteria for Adverse Events v4.0. 

We defined time to gefitinib-induced hepatotoxicity as from the start of treatment with gefitinib to the day that appeared grade 2 or more of hepatotoxicity. We also defined duration of treatment with gefitinib as from the start of treatment with gefitinib to the day that discontinued gefitinib because of gefitinib-induced hepatotoxicity. Retreatment with decreased amount of gefitinib after improvement of hepatotoxicity was performed in some patients. Therefore, duration of treatment with gefitinib included the transient withdrawal period of treatment with gefitinib.

## 3. Results

We identified 127 NSCLC patients who were treated with gefitinib in our institution during an approximately 2-year period. Of these 127 patients, 44 experienced elevated liver aminotransferase levels and 22 (50%) of these 44 switched their treatment from gefitinib to erlotinib. Of these 22 patients, 8 switched their treatment because of gefitinib-induced hepatotoxicity, and the remainder switched because of different problems, for example, disease progression.

These 8 patients (2 men, 6 women) were then evaluated (Tables 1 and 2). Their mean age was 63.0 ± 9.1 years. All patients were histologically diagnosed with adenocarcinoma of the lung. Three patients had WHO performance status (PS) 0-1, and 3 and 2 had PS 2 and 3, respectively; 3 patients had no smoking history. All patients had no liver metastases and negative serologic testing for hepatitis B and C. Five patients had drinking habits, however no patients had history of alcoholic liver injury. EGFR gene mutation was confirmed in 6 patients; the remaining 2 patients were not examined for this parameter. Seven patients were administered 250 mg gefitinib orally once a day and the remainder of 1 patient were administered 250 mg gefitinib orally every other day. All patients achieved disease control by gefitinib, resulting in partial response (PR) and stable disease (SD) in 7 and 1 patients, respectively. Aspartate aminotransferase (AST) and alanine aminotransferase (ALT) levels of all patients were elevated within gefitinib administration and this elevation led to discontinuation of gefitinib. We considered this elevation of AST and ALT as hepatotoxicity caused by gefitinib, because no other reasons, for example, viral hepatitis and alcoholic liver injury were found and discontinuation of gefitinib retrieved the levels of AST and ALT in all patients. Grade 2 and 3 hepatotoxicity was observed in 3 and 5 patients, respectively. The median time to gefitinib-induced hepatotoxicity was 51.5 days from the start of treatment. One patients experienced gefitinib-induced hepatotoxicity within 1 month, while 1 patient experienced it over 6 months later. Retreatment with decreased amount of gefitinib after improvement of hepatotoxicity was performed in 5 patients, however all 5 patients discontinued their treatment with gefitinib because of hepatotoxicity finally. The median duration of gefitinib treatment was 112.5 days. The median duration after hepatotoxicity to withdrawal of gefitinib was 18.5 days.

**Figure 1:**
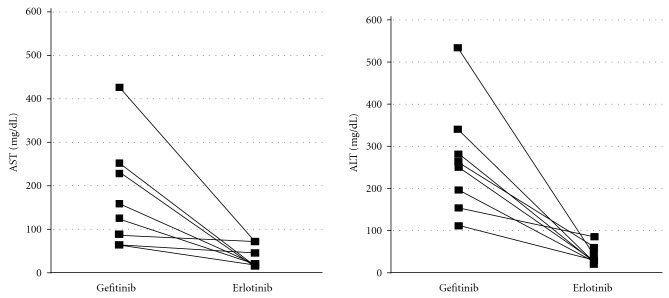
Changes in the peak aspartate aminotransferase (AST) and alanine aminotransferase (ALT) levels following the switch from gefitinib to erlotinib treatment. The levels of both aminotransferases decreased after the change in EGFR-TKIs treatment.

The median duration of treatment with erlotinib was 171.5 days. The initial dose of erlotinib was 150 mg, 100 mg, and 75 mg in 1, 5, and 2 patients, respectively. Two and one patients experienced grade 1 and 2 erlotinib-induced hepatotoxicity, respectively. The change in the peak AST and ALT levels following the switch from gefitinib to erlotinib is indicated in Figure 1. All patients were able to continue erlotinib treatment and none experienced disease progression within the observation period.

## 4. Discussion

Prior to this study, only three case reports have discussed successful treatment of NSCLC patients with erlotinib after gefitinib-induced hepatotoxicity [10–12]. The present study aimed to evaluate whether clinical efficacy was maintaining in patients who switched their treatment from gefitinib to erlotinib due to gefitinib-induced hepatotoxicity, and if this was achieved in the absence of severe erlotinib-induced hepatotoxicity. 

Several studies have reported that EGFR-TKIs induced hepatotoxicity (Table 3). However, the frequency of this adverse effect differed widely between the studies. Thus, in the studies that only included patients with EGFR mutations, the incidence of gefitinib-induced hepatotoxicity was over 50% [5, 6]. In contrast, for studies such as V15-32 and IPASS, where patients were not selected based on EGFR mutation status, the incidence was 24.2% and 9.4%, respectively [13, 14]. The different frequency of gefitinib-induced hepatotoxicity might be attributable to the duration of treatment with gefitinib. The patients with EGFR gene mutation would have a good clinical response and thus continue their treatment with gefitinib for relatively long periods. As a result, they would be susceptible to developing hepatotoxicity. Conversely, the patients without an EGFR mutation would tend to cease gefitinib-treatment within 1-2 months because of reduced efficacy, resulting in a reduced risk of developing hepatotoxicity. 

Several small studies have reported that the disease control rate with erlotinib, when given as a salvage treatment following failure of gefitinib, ranged from 8.7 to 28.6% [15–17]. These results indicate that a substantial proportion of the patients with erlotinib would see a loss in clinical effect when switching to this alternative compound after failure of gefitinib treatment. However, it has been proposed that erlotinib could be used to treat central nervous system metastases that appeared after a good initial response with gefitinib [18, 19]. On the other hand, from the results of previous studies, the incidence of hepatotoxicity due to erlotinib treatment was considered to be relatively low compared with gefitinib [20, 21], and above all, extremely low in the TRUST study [22] (Table 3). In fact, all 5 patients who were reported in three case reports that have discussed successful treatment of NSCLC patients with erlotinib after gefitinib-induced hepatotoxicity [10–12] could continue their treatment with erlotinib which maintained clinical efficacy without severe hepatotoxicity. Erlotinib administration with careful monitoring is thought to be a good alternative strategy for the patients who responded well to gefitinib treatment, but were obliged to discontinue gefitinib because of its hepatotoxicity. 

The differences between gefitinib and erlotinib in terms of liver function sensitivity have not been clarified. In this study, 2 patients were male and 5 patients were smoker. This seems different from the typical patients who are expected to have good response by gefitinib. Male sex and smoking might affect the mechanism of liver injury due to gefitinib and erlotinib. Takeda et al. speculated that gefitinib-induced hepatotoxicity might be caused by an allergic reaction. They also hypothesized that minor differences in the chemical structure or metabolic pathways might also explain the differences with regards to hepatotoxicity [10]. Gefitinib and erlotinib are both metabolized primarily by CYP3A4, CYP3A5, and CYP1A1. CYP2D6 is involved in gefitinib metabolism to a large extent, whereas CYP1A2 is considerably involved in erlotinib metabolism [23]. Kijima et al. proposed the clinical importance of CYP2D6 polymorphism on gefitinib-induced hepatotoxicity [12]. Given its retrospective design and small number of patients examined, the present study had limitations and its results might not entirely reflect the true situation. Further studies are warranted to confirm the results of the present study and to elucidate the mechanism of hepatotoxicity due to EGFR-TKIs.

## 5. Conclusion

In conclusion, erlotinib administration with careful monitoring is thought to be a good alternative strategy for the patients who responded well to gefitinib treatment but which had also resulted in hepatotoxicity.

## Figures and Tables

**Table 1:** Patient characteristics.

Case	Age (y)/sex	Histology	PS	Smoking status	EGFR mutation	Viral hepatitis B/C	Alcoholic liver injury	Liver metastasis	Pretreatment before gefitinib
1	50/F	Ad	2	smoker	positive	−/−	−	−	−
2	51/F	Ad	1	smoker	positive	−/−	−	−	+
3	61/M	Ad	2	non	positive	−/−	−	−	+
4	64/F	Ad	3	smoker	positive	−/−	−	−	−
5	64/F	Ad	1	smoker	unknown	−/−	−	−	+
6	66/F	Ad	2	non	positive	−/−	−	−	+
7	72/M	Ad	3	smoker	unknown	−/−	−	−	−
8	76/F	Ad	0	non	positive	−/−	−	−	+

Abbreviations: Ad: adenocarcinoma; EGFR: epidermal growth factor receptor; PS: performance status.

**Table 2:** Results of hepatotoxicity and treatment with gefitinib and erlotinib.

Case	Worst grade of gefitinib-induced hepatotoxicity	Peak AST/ALT levels by gefitinib-induced hepatotoxicity (mg/dL)	Time to gefitinib-induced hepatotoxicity (days)	Response by gefitinib	Duration of treatment with gefitinib (days)	Retreatment with gefitinib	Initial dose of erlotinib (mg)	Worst grade of erlotinib-induced hepatotoxicity	Peak AST/ALT levels by erlotinib-induced hepatotoxicity (mg/dL)	Response by erlotinib	Duration of treatment with erlotinib (days)
1	2	63/155	22	PR	47	decreased^a^	100	2	46/85	PR	159
2	3	159/280	175	PR	189	decreased^a^	150	0	22/21	SD	92
3	2	128/197	42	PR	42	no	100	0	23/31	SD	184
4	3	89/263	53	PR	204	decreased^a^	100	1	70/62	SD	116
5	3	426/533	55	PR	167	decreased^a^	100	1	71/45	SD	360
6	3	252/252	191	PR	191	no	100	0	17/28	SD	56
7	3	231/342	35	PR	58	decreased^a^	75	0	18/22	PR	304
8	2	62/113	50	SD	50	no	75	0	23/30	SD	412

Abbreviations: AST: aspartate aminotransferase; ALT: alanine aminotransferase; PR: partial response; SD: stable disease.

^a^Retreatment with decreased amount of gefitinib.

**Table 3:** The incidence of hepatotoxicity caused by EGFR-TKIs.

Study name	Incidence of hepatotoxicity Any grade	Grade 3, 4	Ratio of patients with EGFR gene mutation	Ethnicity/race	Number of patients who administrate EGFR-TKIs	References
Gefitinib						

V15-32	24.2%	11.1%	54.%	Japanese	244	Maruyama et al. [13]
IPASS		9.4%	21.7%	Asian	607	Mok et al. [14]
NEJ002	55.3%	26.3%	100%	Japanese	114	Maemondo et al. [5]
WJTOG3405	70.1%	24%		Japanese	177	Mitsudomi et al. [6]

Erlotinib						

Phase II study in Japan	24.2%	3.2%	Not described	Japanese	62	Kubota et al. [20]
OLCSG trial 0705	30%	0%	0%	Japanese	30	Yoshioka et al. [21]
Trust	<1%	<1%	Not described	White, Asian, Black	6580	Reck et al. [22]

Abbreviations: EGFR: epidermal growth factor receptor; TKIs: tyrosine kinase inhibitors.
